# A Rare Presentation of Infective Endocarditis Caused by Streptococcus gordonii Following Transcatheter Aortic Valve Replacement

**DOI:** 10.7759/cureus.76919

**Published:** 2025-01-04

**Authors:** Laith Rhabneh, Todd Cohen, Ali Moosvi, Ahmed M Ashour, Sukrut Dwivedi

**Affiliations:** 1 Internal Medicine, Hackensack Meridian Ocean Medical Center, Brick, USA; 2 Cardiology, Hackensack Meridian Ocean Medical Center, Brick, USA; 3 Internal Medicine, Allegheny Health Network, Pittsburgh, USA; 4 Infectious Diseases, Hackensack Meridian Ocean Medical Center, Brick, USA

**Keywords:** bacterial endocarditides, “infective endocarditis”, prosthetic valve infective endocarditis, streptococcus gordonii, tavr( transcatheter aortic valve replacement)

## Abstract

Infective endocarditis (IE) is an infection of the heart endothelium as well as the heart valve with high mortality rate. The most common cause of infective endocarditis is Staphylococcus aureus. However, IE may be caused by various microorganism depending on the patient risk factor. One of the most important risk factors is the prosthetic heart valve. Patients after transcatheter aortic valve replacement (TAVR) are at risk of IE. The most common organisms to cause IE in TAVR patients are Enterococcus, Staphylococcus aureus, and coagulase-negative staphylococci. The presentation of IE in patients after TAVR is atypical and the conventional ways to diagnose IE have low sensitivity. Therefore the clinician should have high clinical suspicion for it. Herein, we discuss a rare case of infective endocarditis caused by Streptococcus gordonii in a patient who recently underwent TAVR.

## Introduction

Infective endocarditis (IE) is an infection of the heart endothelium as well as the heart valve [1]. The incidence of IE cases is three to 10 cases per 100,000 people per year, with a mortality rate reaching 30% within the first month of infection [2]. In the late 20th century, the average IE patient’s age was in their 40s; however, this has shifted to 70s in recent decades [1]. Historically, IE was linked to dental procedures, this association was proposed due to transient bacteremia that occurs after such interventions [3]. Streptococci, staphylococci, and enterococci were responsible for 80-90% of IE cases. According to the latest studies, the most common cause of IE is Staphylococcus aureus with a prevalence of 26.6% of all cases, followed by viridans group streptococci at 18.7%, other streptococci at 17.5%, and enterococci at 10.5% [2]. Herein, we discuss a rare case of infective endocarditis caused by Streptococcus gordonii in a patient who recently underwent transcatheter aortic valve replacement (TAVR).

## Case presentation

An 84-year-old female presented to the emergency department complaining of lower limb swelling, shortness of breath, and decreased appetite for the past week. Two days prior to her presentation to the emergency department, her cardiologist had increased her diuretic dose, but her symptoms did not improve. The patient denied chest pain, fever, and chills. It is worth mentioning that the patient had undergone aortic valve replacement via TAVR due to severe aortic stenosis in less than two months. Her other medical illnesses included coronary artery disease, atrial fibrillation, hypertension, heart failure with preserved ejection fraction (HFpEF), Meniere's disease, and gout.

On physical examination, her blood pressure was 95/59, heart rate 74 beats per minute, temperature 97.3 °F (36.3 °C), respiratory rate 18 breaths per minute, and oxygen saturation 94% on room air. The cardiovascular examination revealed an irregular rhythm with a systolic murmur, without rubs, or gallops. She also had bilateral rales on lungs auscultation without wheezes, rhonchi or stridor. Extremity examination showed +2 pitting edema of the bilateral lower extremities.

Blood work in the emergency department revealed high white blood cell count (16.0 x 10*3/uL), thrombocytopenia (109 x 10*3/uL), elevated B-type natriuretic peptide (BNP) (839 pg/mL), high sensitivity troponin-I (106 ng/L), procalcitonin (1.03 ng/mL), and lactic acid (3.3 mmol/L). Furthermore, EKG showed rate-controlled atrial fibrillation and Chest X-ray showed mild left pleural effusion and increased pulmonary vascular markings.

**Table 1:** Shows the relevant findings on laboratory investigation along with the reference ranges WBC: white blood cell count; BNP: B-type natriuretic peptide

Parameter	Value	Reference range
WBC	16.0 10*3/uL	4.5 - 11.0 x 10*3/uL
platelets	109 10*3/u	140 - 450 x 10*3/u
BNP	839 pg/mL	0-100 pg/mL
high sensitivity troponin-I	106 ng/L	<34 ng/L
procalcitonin	1.03 ng/mL	<0.50 ng/mL
lactic acid	3.3 mmol/L	0.5 - 1.9 mmol/L

**Figure 1:**
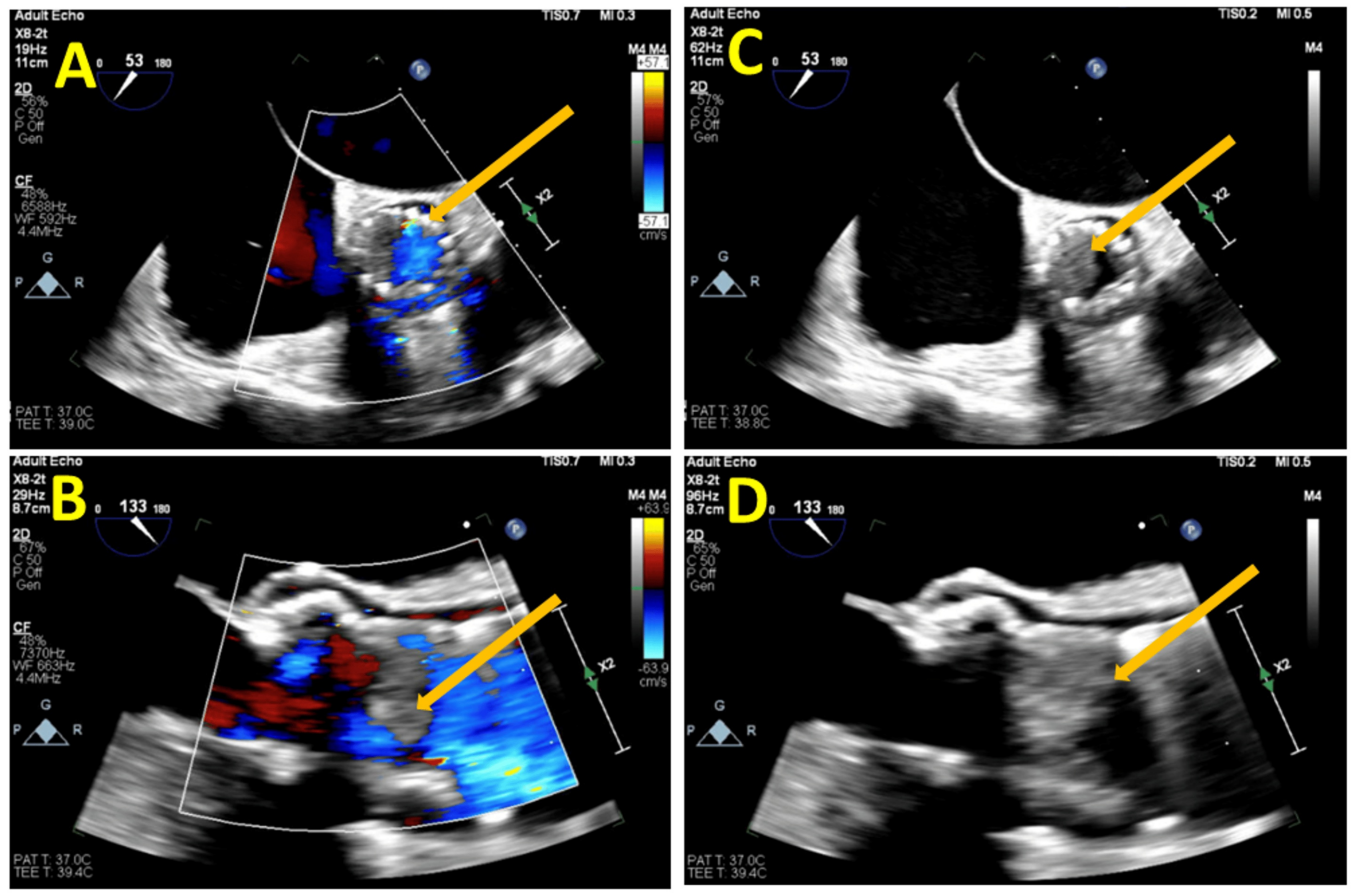
Demonstrating four transesophageal echocardiogram (TEE) images, illustrating the presence of aortic valve vegetation (yellow arrow) A and B demonstrate turbulent blood flow surrounding the vegetation, while C and D depict a large vegetation adhered to the valve.

The patient was admitted to hospital and started on empiric antibiotics (vancomycin and aztreonam). By the following morning, blood culture showed gram-positive cocci, therefore a transthoracic echocardiogram was done and revealed a large, mobile mass (1.6*1.35 cm) on the aortic valve, consistent with vegetation (Figure 1). Blood culture was positive for S. gordonii, and antibiotics were switched to ceftriaxone based on sensitivity analysis. A multidisciplinary team consisting of infectious disease specialists, interventional cardiologists, and cardiac surgeons decided to place a peripherally inserted central catheter (PICC line) for a six-week course of antibiotics and planned to reassess the patient with transesophageal echocardiography (TEE) after completing treatment.

## Discussion

IE affects both native and prosthetic valves, and its course and prognosis depend mainly on patient risk factors and the causative microorganism. Among the most important risk factors for development of IE is rheumatic fever, especially in low income countries. Other risk factors include prior infective endocarditis, congenital heart disease, prosthetic heart valve, advanced age, long-standing steroid use and chronic kidney disease [2].

The indications for treating aortic stenosis with TAVR instead of surgical replacement have expanded to include most patients with moderate to severe aortic stenosis [4]. However, a fatal and serious complication of TAVR is IE, with an incidence of one event per 100 patients per year [4]. Depending on recent study IE after TAVR can be classified into early-onset (occurring within the first two months), intermediate-onset (two to 12 months), and late-onset (>12 months). More than 80% of IE cases occur within the first year of TAVR [5]. Recent studies identify Enterococcus, Staphylococcus aureus, and coagulase-negative staphylococci as the most common pathogens causing IE after TAVR [5].

The case discusses IE caused by Streptococcus gordonii following TAVR. Streptococcus gordonii belongs to the Streptococcus sanguinis group, which is classified as viridans streptococci, along with S. mutans, S. mitis, S. anginosus, S. salivarius, and S. bovis. Streptococcus gordonii is part of the normal flora [6], and can be isolated from the oral cavity, gastrointestinal tract and cutaneous tissue [7]. It can occasionally cause opportunistic infections such as endocarditis, empyema, perihepatic abscesses, pyogenic spondylitis, or spondylodiscitis [7]. In the oral cavity, streptococcus gordonii forms biofilms and dental plaque. Even minor oral manipulations, such as toothbrushing, can release this bacterium into the bloodstream, potentially leading to systemic disease [6].

The presentation of IE after TAVR is unconventional and atypical. The sensitivity of the modified Duke criteria for diagnosing IE after TAVR is approximately 50%, necessitating high clinical vigilance [8]. Recent studies showed that the most common symptom of IE after TAVR is fever, followed by heart failure. Therefore, any patient presenting with signs and symptoms of heart failure after TAVR should be evaluated for IE [5]. 

## Conclusions

This case highlights a rare occurrence of infective endocarditis caused by Streptococcus gordonii in a patient with a recent TAVR. The patient’s presentation with non-specific symptoms such as lower limb edema and shortness of breath, without fever, underscores the atypical nature of post-TAVR IE, where conventional diagnostic criteria like the modified Duke criteria may have limited sensitivity. Prompt recognition of vegetation on the aortic valve through echocardiography and blood cultures confirming S. gordonii facilitated the initiation of targeted antibiotic therapy, which was crucial for a favorable outcome. This case underscores the importance of considering rare pathogens like S. gordonii, part of the viridans streptococci group, in patients with prosthetic valves, particularly following recent dental or oral procedures. It also emphasize the need for high clinical suspicion and prompt diagnostic evaluation when patients present with atypical symptoms, such as heart failure without fever, following TAVR.

Additionally, early identification and targeted antibiotic therapy are critical in managing such cases, as demonstrated by the favorable outcome in this patient. This case underscores the importance of including viridans group streptococci as potential pathogens in TAVR-associated IE and suggests the need for ongoing research into preventive strategies and optimal post-TAVR monitoring protocols.

## References

[1] Cahill TJ, Prendergast BD (2016). Infective endocarditis. Lancet.

[2] Rajani R, Klein JL (2020). Infective endocarditis: a contemporary update. Clin Med (Lond).

[3] Mosailova N, Truong J, Dietrich T, Ashurst J (2019). Streptococcus gordonii: a rare cause of infective endocarditis. Case Rep Infect Dis.

[4] Stortecky S, Heg D, Tueller D (2020). Infective endocarditis after transcatheter aortic valve replacement. J Am Coll Cardiol.

[5] Kuttamperoor F, Yandrapalli S, Siddhamsetti S, Frishman WH, Tang GH (2019). Infectious endocarditis after transcatheter aortic valve replacement: epidemiology and outcomes. Cardiol Rev.

[6] Arbune M, Iancu AV, Lupasteanu G, Vasile MC, Stefanescu V (2021). A challenge of COVID-19: associated infective endocarditis with Streptococcus gordonii in a young immunocompetent patient. Medicina (Kaunas).

[7] Abranches J, Zeng L, Kajfasz JK (2018). Biology of oral Streptococci. Microbiol Spectr.

[8] Zakhour J, Allaw F, Kalash S, Wehbe S, Kanj SS (2023). Infective endocarditis after transcatheter aortic valve replacement: challenges in the diagnosis and management. Pathogens.

